# Surface roughness and profile dataset of Ti6Al4V textured by laser ablation and spark erosion

**DOI:** 10.1016/j.dib.2024.111185

**Published:** 2024-11-30

**Authors:** Victor Caso Moreira, Artur Fernando de Vito, Fabrizio Leonardi, Sergio Delijaicov, Rodrigo Magnabosco

**Affiliations:** Centro Universitário FEI, Avenida Humber de Alencar Castelo Branco, 3972, São Bernardo do Campo, 09850-901 São Paulo, Brazil

**Keywords:** Surface texturing, Surface structuring, Titanium alloy, Interferometer

## Abstract

The Ti6Al4V alloy is widely recognized for its extensive industrial applications, particularly in the aeronautics sector, due to its exceptional strength to-weight ratio and corrosion resistance. In this context, many industrial processes depend critically on surface area, topology, and roughness. A promising approach involves combining Ti6Al4V alloy with polymer composites, which offers significant potential for engineers to design parts that are not only high-performing but also environmentally friendly. Friction stir spot welding (FSSW) emerges as a viable technique for achieving a robust bond between the metal and polymer composite materials. However, a critical factor in this process is the surface profile of the metal, which plays a pivotal role in ensuring strong adhesion between the polymer and the titanium substrate. The data provided focus on analyzing the surface profile and roughness achieved through laser ablation, an advanced technique used for surface texturing, and explores spark erosion as an alternative method.

Specifications Table

**Table:** 

Subject	Mechanical Engineering; Manufacturing Engineering; Surface, Coating and Films.
Specific subject area	Surface Texturing.
Type of data	Raw, Table, Graph and Figure.
Data collection	Surface profiles were acquired using the optical profiler Taylor Hobson CCI MP with green light source - 540 nm wavelength (raw .sur data). They were treated using Gwyddion open-source software, which evaluated roughness parameters and height distributions (.csv). Graphs and Figures (.png) were created using Python.
Data source location	Institution: Mechanical Engineering Department, Polytechnique School, University of São Paulo, 05508-030 São Paulo, Brazil.
Data accessibility	Repository name: Mendelay Data: Surface Roughness and Profile Dataset of Ti6Al4V Textured by Laser Ablation and Spark Erosion Data identification number: doi: 10.17632/8df2swps9b.1 Direct URL to data: https://data.mendeley.com/datasets/8df2swps9b/1
Related research article	None.

## 1 Value of the Data

• Several industrial processes rely heavily on surface area, topology, and roughness. For instance, surface texturing by laser ablation has been used to improve the strength of stir friction welded joints.
• The provided data offers detailed insights into surface profiles generated by both laser texturing and spark erosion, guiding the selection of the most appropriate method and its parameters.
• The study explores various combinations of process parameters, enhancing the understanding of how each factor influences the resulting surface profile.
• The data highlights the critical role of process repetitions in laser texturing, enabling users to estimate the number of cycles required for specific applications.
• This data serves as a valuable resource for researchers and engineers, providing insights that can be applied to the development of customized methods for achieving desired surface characteristics.

## 2 Background

The characteristics of the surface of metallic parts are crucial for various industrial processes as they influence on the occurrence and the rate of chemical reactions, friction, and other phenomena. Recently, surface treatment has gained prominence for enhancing the anchoring effect in dissimilar welds between titanium and polymer matrix composites.

Different approaches can be employed, including the porosity of plates obtained through 3D printing [1], sandblasting [2,3], sanding [4], chemical etching [2,4,5] and laser ablation [[6], [7], [8], [9], [10]]. Each technique provides different surface areas, roughness, and profiles, which should be optimized for the intended application. This work presents the results of surface treatments by laser ablation and spark erosion, with the latter currently being tested by the authors as a strategy to improve friction stir spot welded joints.

## 3 Data Description

Surface profile data was measured, processed, and analyzed for 23 samples: 6 spark-eroded and 17 laser-ablated. Each sample was evaluated in two distinct regions. The spark erosion data covers current ranges of 15–24 A, pulse durations of 200–500 µs, and off-times between 65 % and 80 %. The laser ablation data includes power levels of 15–30 W, frequencies of 30–60 Hz, scanning speeds from 20 to 1000 mm/s, and 1–10 repetitions.

The dataset consists of:

• Raw Surface Profile Data: .sur and .csv files containing the raw surface profile measurements from the interferometer. These files are located in the “Raw Data” folder and named as SampleName Region.sur and SampleName Region.csv, where SampleName corresponds to those in Tables 1 and 2, and Region indicates i or ii (for the first and second regions of each sample).

**Table 1:** Fiber laser parameters for surface texturing.

Sample	Power (W)	Frequency (Hz)	Scanning Speed (mm/s)	Repetitions
LA-01	15	60	500	1
LA-02	15	60	500	5
LA-03	15	60	500	10
LA-04	15	30	20	5
LA-05	15	30	20	10
LA-06	15	60	250	1
LA-07	15	60	250	5
LA-08	21	50	750	1
LA-09	21	50	750	5
LA-10	21	50	750	10
LA-11	21	30	250	5
LA-12	21	30	250	10
LA-13	30	30	1000	1
LA-14	30	30	1000	5
LA-15	30	30	1000	10
LA-16	30	30	30	5
LA-17	30	30	30	10

**Table 2:** Spark erosion parameters for surface texturing.

Sample	Current (A)	Pulse duration (µs)	Off time (%)
SE-1	18	300	75
SE-2	24	500	80
SE-3	24	300	75
SE-4	24	200	65
SE-5	18	200	65
SE-6	15	400	80
• Pre-Treated Surfaces: Surface data that after levelling and bed data treatment, according to *Data pre-treatment*. These files are in the “Treated Surfaces” folder, named SampleName_Region_treated.csv.
• Surface Profiles and 2D Projections: Files containing both the surface profiles and their 2D projections, saved as SampleName_Region_profile.png and SampleName_Region_projection.png in the “Profiles” folder.
• CSV files (SampleName_Region_distribution.csv) stored in the “Height Distribution” folder. These files provide the histograms of measured heights for each region (SampleName_Region_distribution.png).
• CSV files (SampleName_Region_meanprofile.csv) containing the mean profile per region, as described in subsection *Data analysis*. These files are located in the “Mean Profiles” folder. Plots of these profiles are saved as SampleName_Region_meanprofiles.png.
• Parameters Folder: This folder contains roughness data for both linear and areal measurements in the files linear_roughness.csv and area_roughness.csv, respectively. The mean_peaks.csv file includes data on the mean width of eroded lines and the mean teeth-groove height for the laser-ablated samples (detailed in subsection *Data analysis*). Corresponding plots are available in mean_peaks.png. The folder also contains a compilation of the data the linear roughness data for spark eroded samples (comp_se.cvs and comp_se.png).

**Fig. 1:**
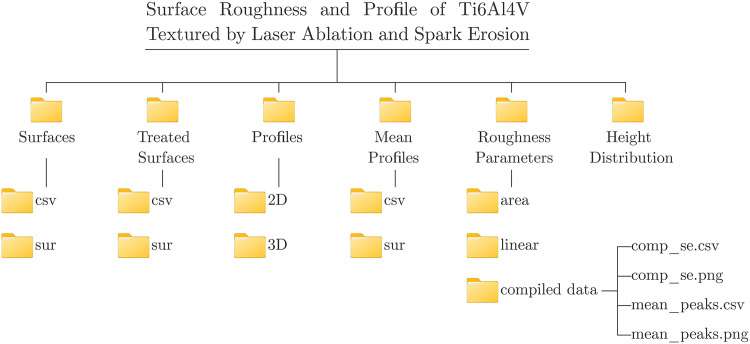
Dataset structure.

The dataset structure is shown in Fig. 1.

## 4 Experimental Design, Materials and Methods

### 4.1 Laser ablation

The laser ablation experiments were conducted using a Ds4 FScan30 fiber laser machine, operating at a wavelength of 1032 nm with a repetition precision of 0.006 mm. Various combinations of laser power (15–30 W), frequency (30–60 Hz), scanning speed (20–1000 mm/s), and repetitions (1–10) were tested, as detailed in Table 1. By systematically varying one or more parameters, the effect of each variable on the surface profile was evaluated.

### 4.2 Spark erosion

Spark erosion was explored as an alternative surface profiling technique. The tests were carried out using an Engemaq EDM 800 NC machine. The primary variables studied were current, pulse duration, and off-time, with their influence on the surface profile assessed according to the experimental plan shown in Table 2. A copper electrode with a diameter of 16 mm was used, and each spark cycle consisted of 5 sparks, followed by periodic withdrawal of the electrode.

### 4.3 Surface observation

Surface profiles were captured using a Taylor Hobson CCI MP optical profiler with a green light source (540 nm wavelength). A 20X magnification lens provided an observation area of 840 × 840 mm^2^, sampled through a 512 × 512 pixel array. Each sample was analyzed in two distinct areas, producing two .sur files per texturing condition.

### 4.4 Data pre-treatment

The raw surface profile data were pre-processed using the Gwyddion open-source software before roughness parameters were calculated and 3D profiles/2D projections were visualized. First, bed data was identified (not measured and disconnected points). The profiles were then leveled using the mean plane subtraction method and the outliers were removed by excluding the 0.5 % higher points. The data was smoothed by a Lagrange interpolation. At last, the leveled and filtered surfaces were standardized by adjusting the minimum height values to zero. The treated .sur files are provided in the dataset repository.

### 4.5 Data analysis

**Fig. 2:**
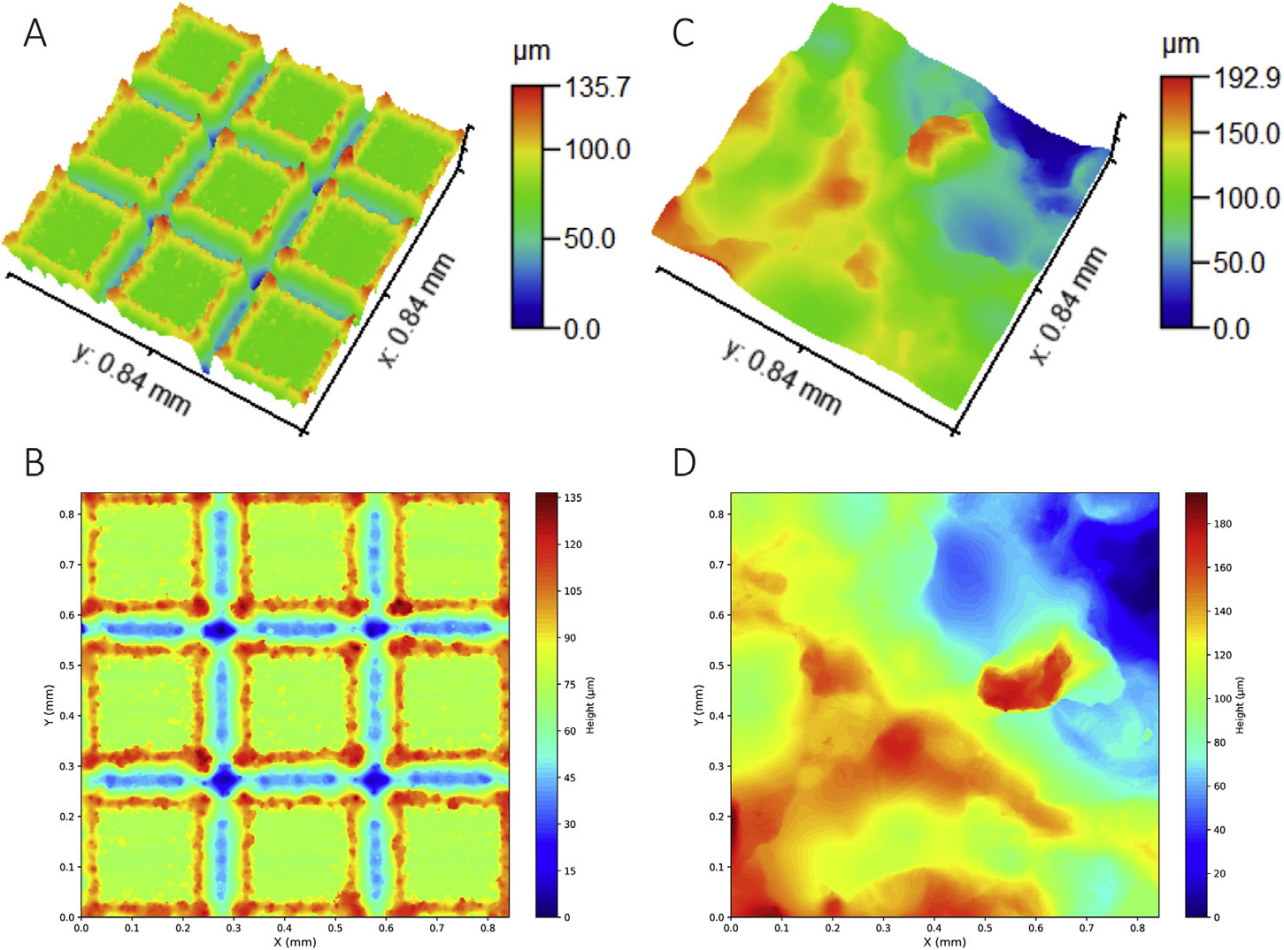
Treated surface: a) and b) laser abladed sample; c) and d) spar erosion sample.

Data analysis was conducted using Gwyddion software, which provided height distribution information and surface roughness parameters such as arithmetic mean height (*S_a_*), root mean square height (*S_q_*), distribution amplitude (*S_z_*), and skewness (*S_sk_*) (as listed in Table 3). Both height distribution and roughness parameters were exported to .csv files. The 3D surface profiles and 2D projections were generated by color-coding the height values for each point (see Fig. 2).

**Table 3:** Example of .csv file for area roughness parameters.

Parameter	Value	Unit
RMS roughness (*S_q_*)	24.5	µm
Mean roughness (*S_a_*)	19.1	µm
Maximum height (*S_z_*)	129	µm
Skewness (*S_sk_*)	0.232

**Fig. 3:**
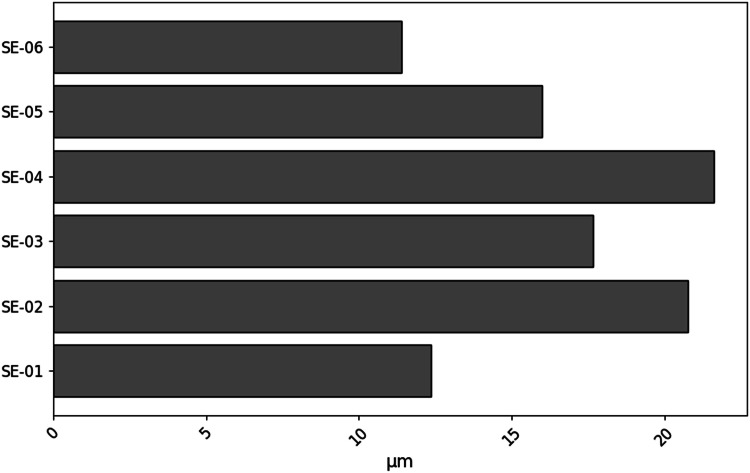
Roughness average (*R_a_*) of the spark erosion samples.

**Fig. 4:**
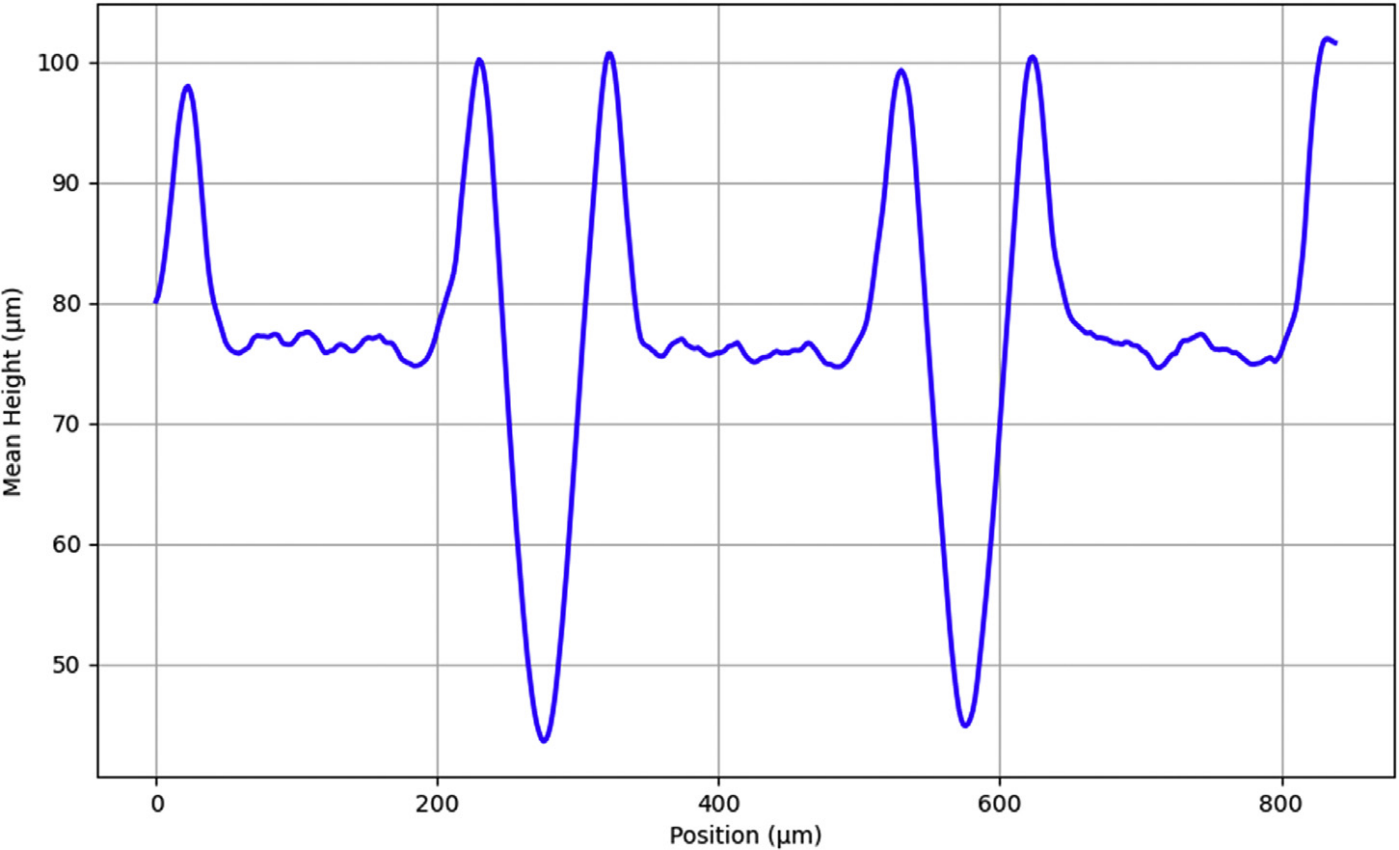
Mean profile of a laser abladed sample (LA-15_ii).

Mean profiles were evaluated by the average of the linear profiles scanned over the vertical axis of the 2d-projections of the surfaces. As the surface data is a 512 × 512 matrix, the mean profile is the average of 512 linear profiles. The corresponding linear roughness parameters (*R_a_, R_q_, R_z_* and *R_sk_*) were exported to .csv files (Table 4). A bar plot (Fig. 3) summarizes the roughness average for the spark-eroded samples. The other roughness parameters behaviors are similar. Additionally, the mean profiles were plotted (Fig. 4).

**Table 4:** Example of .csv file for linear roughness parameters.

Parameter	Value	Unit
Roughness average (*R_a_*)	24.5	µm
RMS roughness (*R_q_*)	19.1	µm
Average maximum height (*R_z_*)	129	µm
Skewness (*R_sk_*)	0.232

**Fig. 5:**
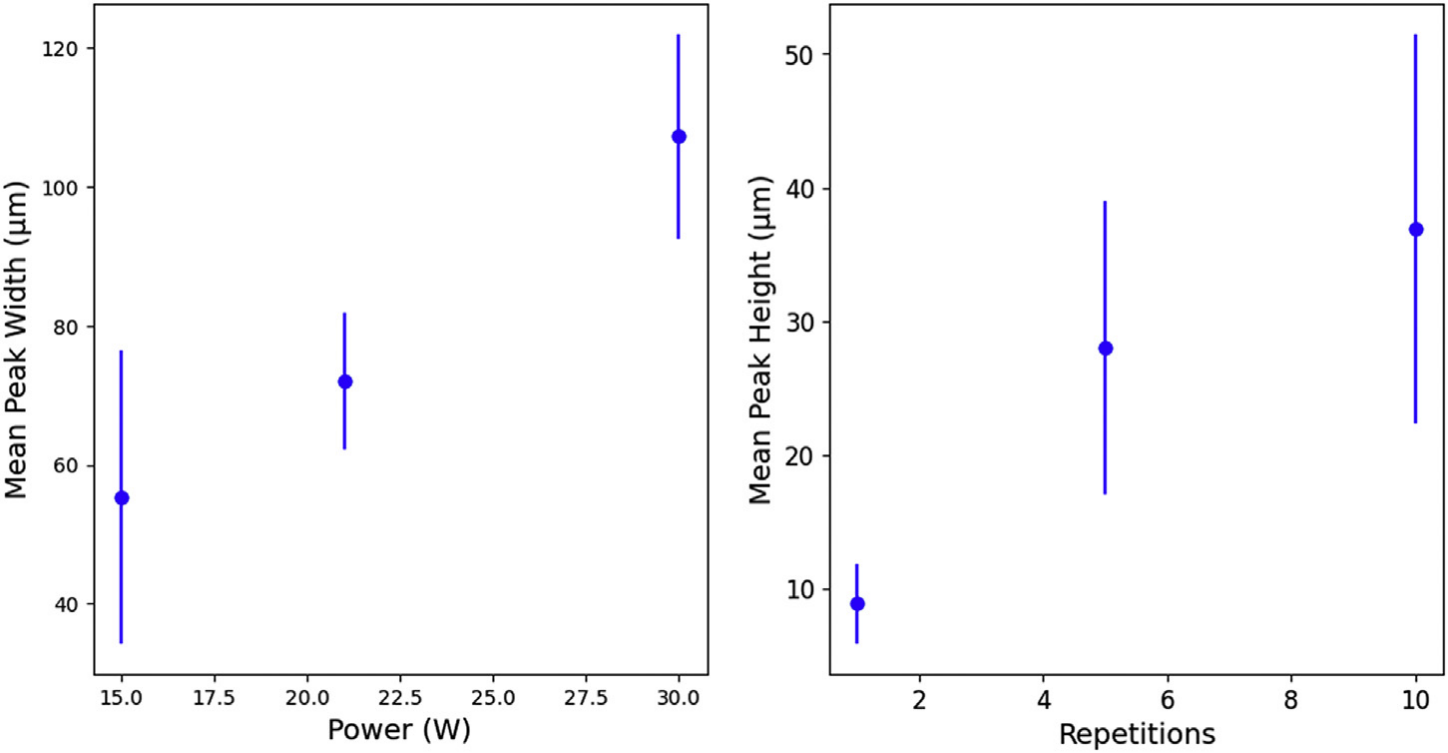
Effect of power and number of repetitions on mean peak width and height, respectively, for laser abladed samples.

For the laser-ablated samples, traditional roughness measurements may not accurately capture the surface characteristics. Instead, the width of the eroded lines in the grid and the maximum height formed at the intersection of these lines (teeth and groove) were measured. This data is available in a .csv file and a grouped plot (.png), summarizing the results. Fig. 5 illustrates the influence of power and number of repetitions on the line width and teeth-groove height. It should be noted that the mean peak heights are underestimated due to its measurement from the mean profile, although the tendency is still valid.

## Limitations

The provided data should serve as a guide for developing surface texturing processes. The exact surface profile may vary depending on the specific equipment used, even when identical parameters are applied. In particular, for spark erosion, factors such as the electrode material, its surface area and cleanliness, the dielectric fluid, and other set variables can significantly influence the resulting profile.

## Ethics Statement

The work did not involve any human subject or animal experiments. The authors have read and follow the ethical requirements for publication in Dat in Brief.

## CRediT authorship contribution statement

**Victor Caso Moreira:** Conceptualization, Methodology, Data curation, Writing – original draft. **Artur Fernando de Vito:** Methodology, Data curation, Writing – review & editing. **Fabrizio Leonardi:** Supervision, Writing – review & editing. **Sergio Delijaicov:** Conceptualization, Supervision, Writing – review & editing. **Rodrigo Magnabosco:** Funding acquisition, Project administration.

## Data Availability

Mendeley DataSurface Roughness and Profile Dataset of Ti6Al4V Textured by Laser Ablation and Spark Erosion (Original data).
